# Coupling functions: dynamical interaction mechanisms in the physical, biological and social sciences

**DOI:** 10.1098/rsta.2019.0039

**Published:** 2019-10-28

**Authors:** Tomislav Stankovski, Tiago Pereira, Peter V. E. McClintock, Aneta Stefanovska

**Affiliations:** 1 Department of Physics, Lancaster University, Lancaster LA1 4YB, UK; 2 Faculty of Medicine, Ss Cyril and Methodius University, Skopje 1000, Macedonia; 3 Department of Mathematics, Imperial College London, London SW7 2AZ, UK; 4 Institute of Mathematical and Computer Sciences, University of Sao Paulo, Sao Carlos 13566-590, Brazil

**Keywords:** coupling functions, coupled oscillators, interactions, dynamical systems

## Abstract

Dynamical systems are widespread, with examples in physics, chemistry, biology, population dynamics, communications, climatology and social science. They are rarely isolated but generally interact with each other. These interactions can be characterized by coupling functions—which contain detailed information about the functional mechanisms underlying the interactions and prescribe the physical rule specifying how each interaction occurs. Coupling functions can be used, not only to understand, but also to control and predict the outcome of the interactions. This theme issue assembles ground-breaking work on coupling functions by leading scientists. After overviewing the field and describing recent advances in the theory, it discusses novel methods for the detection and reconstruction of coupling functions from measured data. It then presents applications in chemistry, neuroscience, cardio-respiratory physiology, climate, electrical engineering and social science. Taken together, the collection summarizes earlier work on coupling functions, reviews recent developments, presents the state of the art, and looks forward to guide the future evolution of the field.

This article is part of the theme issue ‘Coupling functions: dynamical interaction mechanisms in the physical, biological and social sciences’.

## 1. Introduction

A coupling function describes the physical rule specifying how an interaction occurs. Being directly connected with functional dependences, coupling functions focus not so much on *whether* interactions exist, but more on *how* they appear and develop. For instance, the magnitude of the phase coupling function affects the oscillatory frequency directly and describes how the oscillations are being either accelerated or decelerated by the influence of the other oscillator. Similarly, if one considers the amplitude dynamics of interacting dynamical systems, the magnitude of the coupling function will prescribe how the amplitude is increased or decreased by the interaction.

A coupling function can be described in terms of its *strength* and *form*. The coupling strength, already a relatively well-studied quantity, describes the magnitude and the extent of the coupling relationship, whereas the form of the coupling function adds a separate dimension and perspective probing directly the mechanisms of interaction. In other words, the mechanism is defined by the functional form giving the rule and process through which the input values are translated into output values—how the input influence from one system is translated into the output effect on the other system to which it is coupled. Thus the coupling function can describe qualitative transitions between distinct states of the systems e.g. routes into and out of synchronization. Depending on the known form of the coupling function and the quantitative inputs, one can predict, control or engineer the transitions to/from synchronization. Decomposition of a coupling function provides a description of the functional contribution from each separate subsystem within the coupling relationship. Hence, the use of coupling functions amounts to revealing the mechanisms underlying the functionality of the interactions.

Close attention has recently been devoted to the study of coupling functions, treating their different aspects, including theory, methods and applications. It is already a significant body of work highlighting the universal meaning of coupling functions for interacting dynamical systems, quite generally. In effect, a new scientific subfield dedicated to the study of coupling functions has started to emerge, paving the way to specialized scientific gatherings on coupling functions in the form of minisymposia, workshops and conferences, as well as to dedicated reviews. Thus the topical issue has emerged naturally to unite and document these activities in one place, thereby bringing out the universality of the coupling function as a theoretical construct.

The need for a theme issue is perhaps most strongly supported by the fact that the applications of coupling functions span such a great diversity of very different scientific areas. Although originally stemming from physics and mathematics, applications have now emerged in, for example, chemistry, neuroscience, physiology, system microbiology, mechanics, climate science, secure communications and the social sciences. The experts in these separate fields seldom encounter each other or come across work on coupling functions by scientists in the other areas. Nor are they exposed to the larger picture in which the general relevance and universality of coupling functions becomes clear. Furthermore, the mutual support that could assist accelerated progress is mostly absent, and there is obviously a danger of unintended rediscovery. One of our aims is therefore to pull together and integrate existing knowledge about coupling functions from the widely-scattered specialist research publications where it currently appears. The theory and the methods will be covered by experts from physics and applied mathematics, and these will then be applied to electrochemical, neural, cardiorespiratory, climate, electrical engineering and social interactions.

The majority of the publications on methods for reconstruction of coupling functions have appeared during the last decade. The work was made possible by the availability of enlarged computing facilities, enabling the application of advanced theoretical approaches to create new ways of reconstructing coupling functions from real data. The latter have been measured from widely differing interacting systems. These developments have led to convenient ways of using coupling functions to better understand, control, predict, and engineer the interactions in the areas of interest.

A recent review [1] of coupling functions has covered the basic concepts—including the theory, methods and applications related to some of the more important earlier works in the area. The present theme issue is complementary, helping to move the field forward. In particular, it extends and updates that review by reporting subsequent developments and research directions in the field. It also provides an opportunity for leading experts to report their latest results and to express their own opinions and viewpoints.

## 2. Recent works on coupling functions

The evolution in our understanding of coupling functions has been tightly linked to attempts to detect and formulate theoretically the nature of dynamical systems, oscillations and interactions in particular areas, including especially physics, biology, chemistry and climate [2–9].

Recent theoretical progress has tended to concentrate on particular aspects of coupling functions, with some being more focused on the overall interactions or qualitative states, while others study unique characteristics of coupling functions and how they affect the overall interactions [10–16]. In this way, the coupling functions play important roles in the phenomena and the qualitative states resulting from the interactions. Examples include synchronization [9,17–19], amplitude and oscillation death [20–23] and the low-dimensional dynamics of ensembles [24–26]. Much attention has also been devoted to coupling functions and networks [10,12,13,27,28]. Coupling functions can also have important implications if used in a non-traditional way like, for example, ones that are of biharmonic form or non-pairwise coupling functions [10,11,29,30]. Particularly interesting is a study that defines design strategies for coupling functions in order for the systems to achieve a state of generalized synchronization [31]. This recent work is of particular interest in that it treats the full state-space dynamical system, and not just the approximative phase dynamics. A similar approach was used in phase dynamics previously, and the procedure was presented as synchronization engineering [32,33].

**Figure 1.**
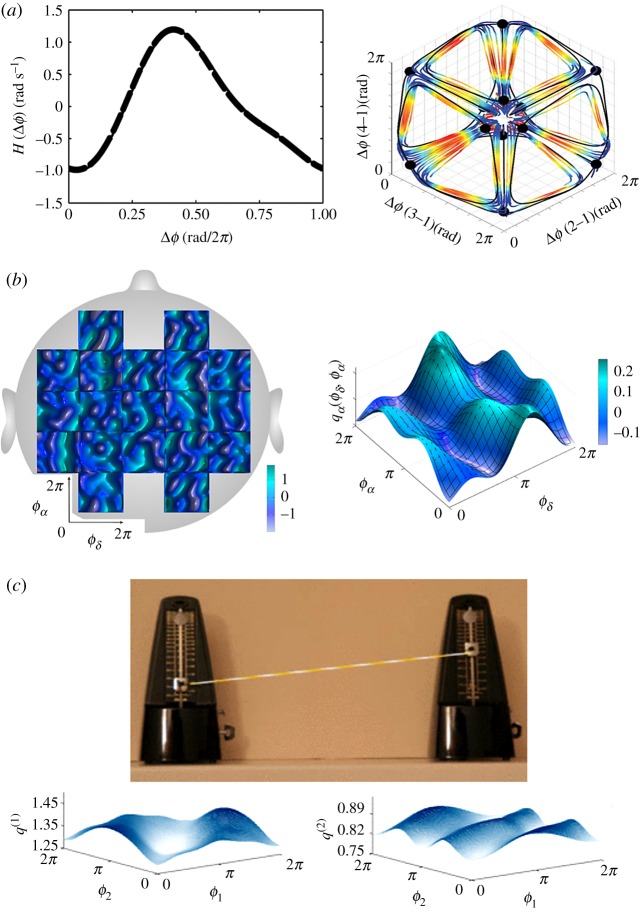
Examples of coupling functions in a diversity of applications. (*a*) In chemistry, a system of four non-identical electrochemical oscillators has been engineered [32], using a specific coupling function to generate sequential cluster patterns: on the left is the optimized target coupling function, and on the right the corresponding trajectories in state space during slow switching. (*b*) In neuroscience, cross-frequency *δ*-*α* neural coupling functions [46] showing the spatial distribution over the head and the averaged *δ*-*α* coupling functions. (*c*) In mechanics, bidirectional coupling functions for a pair of metronomes coupled with a rubber band [47].

The recent development of powerful methods for reconstruction of coupling functions from measured data has allowed a linkage between the theory and the methods concerned, offering opportunities to investigate many real experimental systems and their interactions [34–45]. These methods have mediated applications, not only in different subfields of physics and mathematics, but also in quite different scientific fields. Figure 1 illustrates a few examples. Reconstruction within the new methods is based on a range of techniques for the inference of dynamical systems, including least-squares fitting, kernel smoothing, Bayesian inference, maximum-likelihood (multiple-shooting) methods, differential evolution, stochastic modelling and phase resetting [34–38,44,45,48,49].

Applications have now been reported in, for example: chemistry, neuroscience, cardiorespi- ratory physiology, biology, social sciences, mechanics, ferromagnetism, secure encryption and ecology [28,32,35–37,46,47,50–61]. In chemistry, coupling function methods have been used for understanding, effecting, and predicting interactions between oscillatory electrochemical reactions [28,32,37,56,57]. In biology the new methods have been applied to characterize genetic oscillators and homogeneous flocking [51,52], and in cardiorespiratory physiology they have been used for reconstruction of the human cardiorespiratory coupling function and phase resetting curve [35,36,59,62]. In social sciences, the function underlying the interactions between different social and economic dynamical dependences has been determined [50,51,60]. The mechanical coupling functions between coupled metronomes were reconstructed in a similar way [47]. A new protocol based on state-space coupling functions has been developed for secure communications [61,63]. Coupling functions have also been used to study coupled oscillating magnetizations [53,54].

Arguably, the greatest current interest is coming from neuroscience. This may be because the brain is a highly-connected complex system [64], with connections on different levels and dimensions, many of them carrying important implications for characteristic neural states and diseases. Coupling functions are particularly appealing here because they can characterize the particular mechanisms behind these connections. Recent works have encompassed the theory and inference of a diversity of neural phenomena, levels, physical regions, and physiological conditions [45,65–75].

## 3. The roadmap of the issue

The contributors have been chosen so as to match the roadmap of the theme issue, which is organized around three main pillars:

— Theory
— Methods
— Applications

It must be emphasized, however, that there are no hard borderlines so that topics often rely on support from more than one pillar. For example, although some contributions relate predominantly to theory, or to methods, they also carry important implications for the applications of coupling functions, and *vice versa*.

### (a) Contributions to theory

The theoretical contributions are developed sequentially in order of increasing complexity, starting from basic formulations and moving on towards new applications. To set the context, this part starts with the review article by Kuramoto & Nakao [76] on the concept of dynamical reduction theory for coupled oscillators. Their approach places particular emphasis on the remarkable structural similarity that exists between centre-manifold reduction and phase reduction methods. Rosenblum and Pikovsky then generalize the notion of the phase coupling function for the nonlinear case [77], going beyond the usual first-order approximation in the strength of the force, and they illustrate the idea by application to a paradigmatic oscillator model. The theory of weak coupling for neuroscientific applications is reviewed by Ermentrout *et al.* [78], who consider non-smooth systems and introduce the idea of isostable reduction to explore behaviours beyond the weak coupling paradigm. Ashwin *et al*. consider the effective network interactions and dynamical behaviour that arise in cases where the coupling function between oscillatory units has ‘dead zones’ [79].

### (b) Contributions on methods

Contributions to the methods part first outline the methodological framework in terms of effective connectivity, and then provide a comprehensive discussion and comparison of the most important coupling function methods used in physics. There are also discussions about information transfer across timescales and the importance of surrogate testing for coupling functions. Some of the methods are demonstrated on example applications. Jafarian *et al*. provide a brief history of dynamic causal modelling, focusing on the Bayesian reduction of state space models of coupled systems [80]. They illustrate the usefulness of these techniques by modelling the neurovascular coupling. Tokuda *et al*. use a practical method to describe the process of estimating coupling functions from data obtained from complex dynamical systems [81], and demonstrate its benefits on experimental data from a forced Van der Pol electric circuit. Rosenblum *et al*. present a method for dynamical disentanglement of the phase dynamics of oscillatory systems [82], and apply it to cardio-respiratory interactions to reconstruct the respiratory sinus arrhythmia. Paluš [83] describes a methodology for the detection of cross-scale causal interactions, based on wavelet decomposition in terms of instantaneous phases and amplitudes, and an information-theoretic formulation of Granger causality combined with surrogate data testing. The methodology is then applied to climate data, for analysis of interactions and information transfer in the dynamics of the *El Niño* Southern Oscillation. Needless to say, these methodological works also have applications, so there is partial overlap with the following section on applications.

### (c) Contributions on applications

The applications part presents five applications of the methods to interaction data drawn from chemistry, physiology, neuroscience, climate, electrical engineering and social sciences. In electrochemistry, Sebek *et al*. [84] demonstrate anti-phase synchronization of collective dynamics with intrinsic in-phase coupling of two groups of electrochemical oscillators. They showed how theory predicts that, for anti-phase collective synchronization, there must be a minimum internal phase difference for a given shift in the phase coupling function. As an example in neuroscience and cardiorespiratory physiology, Hagos *et al*. discuss [85] how time-variability in the form of the neural and cardiorespiratory coupling functions can trigger qualitative transitions in the interactions. Through numerics and theoretical considerations, they show how time-varying forms of coupling function can induce synchronization transitions, even when the net coupling strength is time-invariant. Dolenc *et al.* write about an electrical engineering example, showing how an analysis of coupling functions in solid oxide fuel cells can assess and follow the slow evolution of the degradation dynamics [86]. Moon & Wettlaufer [87] discuss the application of coupling functions to archived climate data, considering a range of timescales and focusing on air–sea interactions in tropical oceans. They construct the relevant coupling functions between two tropical climate indices—the El Niño/Southern Oscillation (ENSO) and the Indian Ocean Dipole (IOD)—to interpret the mutual interactions between these two air–sea interaction phenomena in the Pacific and Indian Oceans. Taking our final example from social science, Blomqvist *et al.* present a technique based on Gaussian processes for inferring the dynamics of a rising radical right-wing political party [88].

## 4. Conclusion

We hope that readers will find this theme issue interesting, and that it can serve as a useful starting point for those new to the area who discover, or suspect, that there will be benefits from coupling functions in their work. The topics are covered by leading experts, and each contribution has an ample and up-to-date bibliography. The collection is also comprehensive in the sense that it spans the theory, methods and applications of coupling functions. We anticipate that this will foster further integration between the three aspects, perhaps leading to new developments of coupling functions that are as yet unseen.

## Data accessibility

This article has no additional data.
